# Unusual Presentation of Central Diabetes Insipidus in a Patient With Neurosarcoidosis

**DOI:** 10.1177/2324709616667511

**Published:** 2016-09-09

**Authors:** Vedha Sanghi, Aanchal Kapoor

**Affiliations:** 1 Department of Pulmonary and Critical Care Medicine, Cleveland Clinic, Cleveland, OH, USA

**Keywords:** neurosarcoidosis, thyroxine, hypernatremia, central diabetes insipidus, oliguria, hypothyroid, hypothalamic pituitary, altered mental status

## Abstract

Hypernatremia is a frequent cause of intensive care unit admission. The patient presented in this article had hypernatremia refractory to D5W (dextrose 5% water) therapy, which led to a complex investigation. Workup revealed central diabetes insipidus most likely secondary to flare up of neurosarcoidosis. The challenge in terms of diagnosis was a presentation with low urine output in the setting of hypernatremia resistant to treatment with desmopressin. This case unfolded the role of hypothyroidism causing secondary renal dysfunction and hence needed continued treatment with thyroxine in addition to treatment for hypernatremia.

## Introduction

Sarcoidosis is a multisystem noncaseating granuloma-forming disorder with a debate between unknown etiology and immune-mediated.^1-3^ Central nervous system involvement is seen in <10% of sarcoidosis patients.^1^ The hypothalamic-pituitary axes can be affected leading to neuroendocrine manifestations, such as diabetes insipidus (DI). Panhypopituitarism is the initial presenting complaint in 2%.^3^ The clinical course depends on the initial localization, and presentation can be monophasic, relapsing-remitting, or progressive with intermittent episodic deteriorations.^1^

We describe a case of neurosarcoidosis-induced panhypopituitarism manifesting as central diabetes insipidus (CDI) in which subclinical hypothyroidism masked the classic presentation of polyuria. The highlight of this case report is the reversible nature of renal dysfunction after replacing thyroid hormone deficiency.^4^ This documentation is made to add clinical evidence to existing knowledge while interpreting similar observations.

## Case Presentation

A 40-year-old female presented to the emergency department with altered mental status, slurred speech, and a possible episode of seizure after she was found soiled in urine and feces. Her past medical history was significant for systemic lupus erythematosus limited to the skin manifestations and an unspecified seizure disorder for which she was taking anti-epileptics. She got diagnosed with pulmonary sarcoidosis 6 years ago in conjunction with central nervous system involvement for which she was on immunosuppression with methotrexate, infliximab, and prednisone. She had a significant history of being noncompliant with her medications. She had a history of traumatic brain injury, left hip replacement for avascular necrosis, and a recent open reduction internal fixation of the right tibial fracture. Family history was noncontributory. There was no history of substance abuse, sick contacts, travel, or animal exposure. Vital signs at the time of admission were blood pressure 104/66 mm Hg, pulse rate 97 beats per minute, respiratory rate 24 breaths per minute, and temperature 36.7°C. She was confused and disoriented and had dry mucus membranes. Physical examination including neurological assessment was unremarkable.

### Assessment

**Figure 1.**
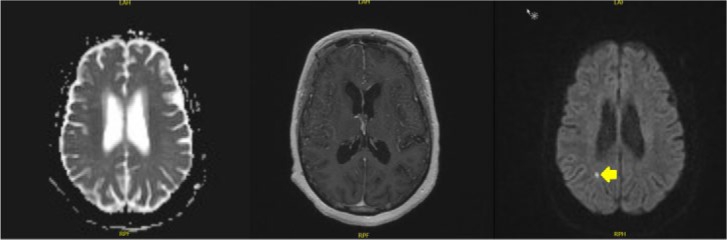
MRI at admission. Marked improvement in leptomeningeal/pial enhancement with mild residual enhancement (in the posterior fossa). Mild diffuse volume loss with stable prominence of the ventricular system. Five millimeter punctate focus of white matter restricted diffusion in the right parietal lobe.

**Figure 2.**
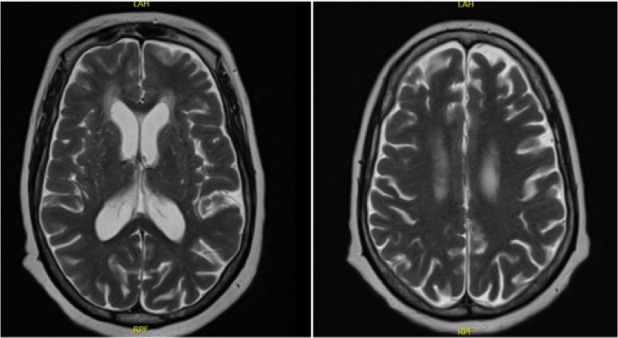
MRI from previous visit. Prominent pial enhancement throughout the brain, more in the right cerebral hemisphere with adjacent cortical FLAIR changes.

The initial assessment focused on etiology of altered mental status, which we attributed to urinary tract infection based on urine analysis. Differentials included postictal stage as levetiracetam level was below therapeutic range or flare up of neurosarcoidosis. Empiric therapy with vancomycin and piperacillin-tazobactam was started while awaiting cultures. Levetiracetam 250 mg BID was also begun. Continuous bedside electroencephalogram for 72 hours showed intermittent rhythmic slow generalized bifrontal activity, suggestive of mild diffuse encephalopathy and negative for epileptogenic focus. Magnetic resonance imaging (MRI; Figure 1) of the brain showed an improvement in the previously established leptomeningeal/pial enhancement with mild residual enhancement particularly in the posterior fossa when compared to the MRI (Figure 2) from 2 months ago. It also showed new punctate 5-mm right parietal infarctions.

Over the course of next 5 days, her sodium level started to rise, from 138 (Day 1) → 149 (Day 2) → 159 (Day 3) → 170 (Day 4) → 169 (Day 5) and did not respond to D5W (dextrose 5% water) administration (Table 1). The dangerously rising sodium levels warranted medical intensive care unit admission. Urinalysis showed urine sodium of 48 mEq/L, and urine osmolality was 113 mOsm/kg. Serum osmolality was 335 mOsm/kg, consistent with a diagnosis of DI. Interestingly, urine output (Table 2) remained consistently low, seeming that 100 cc/h was her best polyuric response, thus questioning the diagnosis of DI. A DDAVP stimulation test (Table 3) was performed, that confirmed the diagnosis of CDI, with a >100% increase in urine osmolality and >60 mOsm/h excretion of sodium. A pituitary enzyme panel to evaluate other parts of axes was done: LH (luteinizing hormone) = 4.8 (normal = 1-25 mU/mL), FSH (follicle-stimulating hormone) = 6.1 (normal = 4.7-21.5 mU/mL), prolactin = 23.2 (normal = 2.0-17.4 ng/mL), fT4 = 0.6 (normal = 0.7-1.8 ng/dL), TSH (thyroid-stimulating hormone) = 2.340 (normal = 0.400-5.500 µU/mL), IGF-I (insulin-like growth factor-1) = 61 (normal = 93-278 ng/mL), early morning cortisol = 26 (normal = 3.4-26.9 µg/dL), ACTH (adrenocorticotropic hormone) = 19 (normal = 9-52 pg/mL).

**Table 1.** Serum Sodium Levels.

	Day 1	Day 2	Day 3	Day 4	Day 5	Day 6	Day 7	Day 8	Day 9	Day 10	Day 11	Day 12	Day 13	Day 14	Day 15	Day 16
Serum sodium levels (mmol/L)	138	149	159	170	168	165	163	152	144	147	139	146	147	149	147	145

**Figure:**
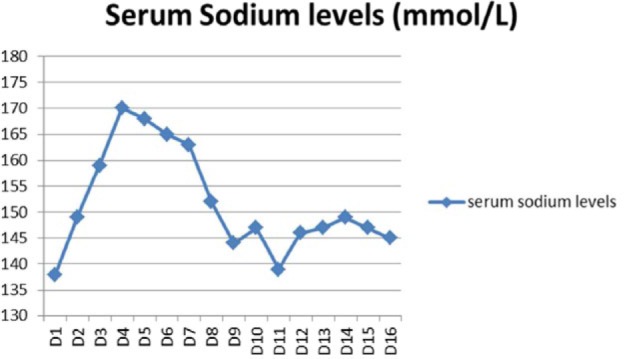


**Table 2.** Total Fluid Input, Output, and Net.

	Day 1	Day 2	Day 3	Day 4	Day 5	Day 6	Day 7	Day 8	Day 9	Day 10	Day 11	Day 12	Day 13	Day 14	Day 15	Day 16
Total intake (mL)	1400	3022	2416	3171	2857	960	1583	4239	2905	270	890	240	480	570	633	450
Total output (mL)	0	0	300	0	1840	2295	1500	3090	2550	2275	775	1865	1862	723	602	1104
Net intake/output (mL)	1400	3022	2116	3171	1017	−1335	83	1149	355	−2005	115	−1625	−1382	−153	31	−654

**Figure:**
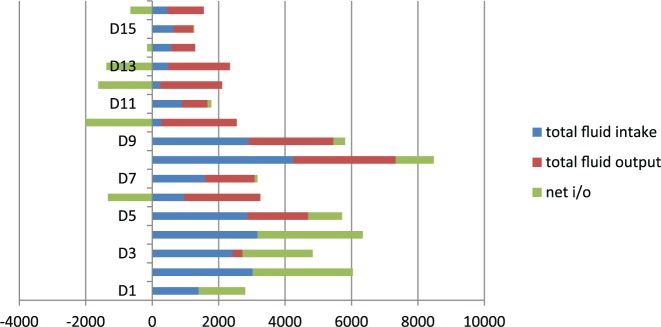


**Table 3.** DDAVP Stimulation Test.

Urine sodium	48 mEq/L	Desmopressin 2 µg IV given	96 mEq/L
Urine osmolality	118 mOsm		346 mOsm

**Figure 3.**
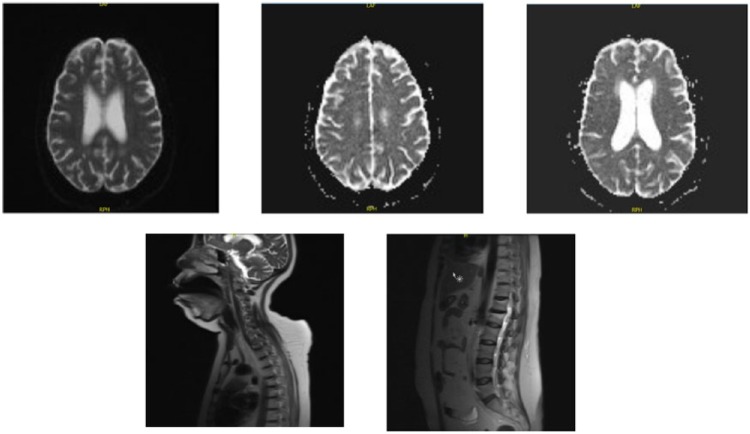
MRI at diagnosis. MRI Brain: diffuse extensive leptomeningeal enhancement with components of nodularity. Additional punctate foci of enhancement involve the brain parenchyma. Some associated high T2 and FLAIR signal at corresponding locations. Additional patchy areas of high T2 and FLAIR signal involving periventricular white matter, particularly about the frontal horns. MRI Cervical Cord: Subtle punctate nodular foci of enhancement along the cervical cord surface, patchy high T2 signal involves the upper cervical cord extending to the C2 level. MRI Thoracic Cord: Small nodular punctate foci of enhancement along the thoracic cord surface.

The etiology of DI was thought to be either secondary to new infarct or due to neurosarcoidosis. A repeat MRI (Figure 3) focusing on pituitary was done. It showed extensive leptomeningeal enhancement in the supra- and infratentorial compartments with components of nodularity and scattering throughout the surface of the cervicothoracic spinal cord. This enhancement was increased as compared to the MRI taken at admission and was most consistent with neurosarcoidosis flare.

### Management

She was started on DDAVP and continued on D5W correction. However, high sodium level and low urine output continued. At this point, we considered the possibility that central hypothyroidism from pituitary dysfunction was confounding the symptoms, and levothyroxine replacement with 75 µg OD ultimately normalized sodium levels to a range of 139 to 145 mmol/L.

After consultation with neurology/rheumatology teams, she was started on pulse dose intravenous methylprednisone 1 g daily for 3 days followed by a gradual taper for neurosarcoidosis flare. Her mental status improved significantly, and she was discharged home on levothyroxine and prednisone with a planned follow-up and recheck on sodium levels.

## Discussion

This is a previously unreported case of neurosarcoidosis induced panhypopituitarism manifesting with hypernatremia as a result of CDI in which subclinical hypothyroidism masked the classic presentation of polyuria. The challenge, in this case, is because of underrecognition in clinical practice. Similar to our patient, Non et al^2^ reported a patient who presented with neurosarcoidosis-induced CDI, whose initial presentation was with nausea, vomiting, and dehydration. CDI manifestations were masked by the presence of secondary adrenal insufficiency and was uncovered only after few days of treatment with steroids when she started to develop increased thirst, polyuria, and hypernatremia. At that time investigation revealed CDI with an appropriate response to treatment with desmopressin. In comparison, our patient was initially resistant to improvement following desmopressin administration and slowly unmasked the presence of subclinical hypothyroidism. These 2 cases highlight that the pathophysiology involved is impaired renal free water clearance, and we see the significant role that thyroxine and corticosterone, the endocrine hormones, play in the proper functioning of the kidney.

The incidence of neurosarcoidosis is 0.2/100 000.^2^ In a recent cross-sectional study^5^ of 52 patients with sarcoidosis, it was found that neurologic symptoms associated with sensory impairment (71%) was the first clinical manifestation, followed by chronic aseptic meningitis (37%), cranial nerve palsy (31%), and spinal cord involvement (23%). Facial nerve palsy was the most commonly involved cranial nerve palsy. Neuroendocrine involvement (6%) with panhypopituitarism (4%) or DI (2%) was the least common presentation. Systemic involvement (79%) was highest for lung (67%), followed by eye (33%). Only 1% of patients had a history of sarcoidosis and autoimmune disease. Response to therapy with prednisone showed improvement (67%), and the most common complication during follow-up was the side effects of prednisone (87%).

The hypothalamic thyrotropin releasing hormone regulates the secretion of thyroid-stimulating hormone from the anterior pituitary gland, which further influences the thyroid gland to produce tetraiodothyronine and triiodothyronine,^6^ the 2 hormones known to play a significant role in the metabolic processes of the human body. The mechanism of action of thyroid hormones involves entry of hormone receptor complex into the nucleus of the target cell, activating the gene for synthesis and translocation of proteins.^7^ A change in the circulating serum levels of these hormones can affect the target organ in many ways.^6,8^ In our patient, we describe one such significant effect on the kidney.

Thyroid hormones have been reported to affect the growth and function of a kidney from as early as the perinatal period, and a deficiency can result in decreased growth size.^7^ Hypothyroidism decreases the kidney-to-body weight ratio.^9^ There is thickening of the glomerular basement membrane, expansion of mesangial matrix,^10^ decreased glomerular surface area, decreased filtration pressure, and a loss of mass in thick ascending limb, proximal and distal convoluted tubules of the nephron^4,6,9^ However, the structural changes are reversible after treatment with thyroid hormone supplementation, which almost doubles the renal mass.^9^

Hypothyroidism affects kidney function in many different ways. It decreases the renal plasma flow (RPF) and glomerular filtration rate (GFR) via prerenal or systemic and intrinsic renal dysfunction or by direct effects of thyroid hormones on the kidney respectively.^4,7,9^

Systemically, hypothyroidism causes dramatic hemodynamic effects due to its effect on the cardiovascular system. There is a drop in cardiac output resulting in reduced renal blood flow and the glomerular transcapillary hydrostatic pressure.^4,6^ The cardiac changes that are responsible are bradycardia, decreased cardiac contractility, increased systemic vascular resistance,^7,11,12^ and diastolic hypertension with narrowed pulse pressure.^8^ The vascular changes that are responsible are the effect on the vascular smooth muscle tone, its reactivity, and an increase in nitric oxide synthase activity in the heart, kidney, and large blood vessels,^4,12^ which result in increased intrarenal vasoconstriction, reduced renal response to vasodilators, and reduced expression of renal vasodilators such as vascular endothelial growth factor and insulin like growth factor-1.^6,13^

Directly, hypothyroidism causes an approximately 40% fall in GFR^14^ due to its regulation of the adrenergic and dopaminergic receptors on the renal tubular cells,^15^ which have an effect on the renin-angiotensin-aldosterone axis, renin release, and angiotensinase activity.^7,16^ The low GFR results in an increase in serum creatinine levels.^4^ Another study^6^ noted a fall in serum creatinine after thyroid hormone replacement along with an increase in GFR and estimated RPF. There is rapid normalization in creatinine levels with thyroid replacement after short periods of hypothyroidism but slower and incomplete recovery after prolonged periods of hypothyroidism.^17^ This shows the significance of early detection in patients who may benefit from a complete recovery.

Thyroid hormone also affects the renal tubular function by influencing the expression and activity of ion transporters^6^ such as Na^+^-K^+^ cotransporter, Na^+^-H^+^ exchanger, Na^+^/K^+^ ATPase, chloride ion transport channel, Na^+^-Ca^2+^ exchanger, Na^+^-HCO_3_^−^ exchanger, Na^+^-Pi cotransporter, Na^+^-K^+^-2Cl^−^ cotransporter, and enzymes of mitochondrial energy metabolism in the proximal convoluted tubule.^6,9^ As a result, the secretory and reabsorptive processes of major ions such as sodium,^8,9^ potassium,^6,9^ bicarbonate,^9^ chloride,^9^ and calcium^7^ are impaired.

In hypothyroidism, there is a fall in sodium and bicarbonate tubular reabsorption and impaired regulation of the expression of aquaporin 1 and 2 channels that are responsible for free water clearance in the distal tubules.^3,6,18^ The result is a loss of medullary hypertonicity and the ability to concentrate urine.^19^ There is a reversible increase in the sensitivity to ADH (anti diuretic hormone) at the collecting ducts that increases free water absorption. The increased fluid retention is unable to suppress both ADH and pituitary resistance resulting in further free water retention.^20^ There is also increased non-osmotic ADH secretion, from low cardiac output stimulating the carotid baroreceptors.^6,21^ These mechanisms clearly explain the presentation of oliguria in our patient despite confirmed diagnosis of DI and no response to desmopressin that improved following thyroxine administration.

Although the effect of thyroid hormone on renal function in subclinical hypothyroidism has not been extensively studied,^8^ there is enough available evidence showing that thyroid hormones play a significant role in influencing the renal function of water and electrolyte balance in the body.

## Conclusion

The reversible changes that occur in the nephrons of the kidney due to decreased level of thyroid hormones can affect the classic presentation of a disease process, such as polyuria in DI and challenge the diagnosis of a common case. This patient despite having a proven diagnosis of CDI and receiving appropriate treatment with desmopressin did not show any improvement in the serum sodium and mental status levels. After a thorough review of the literature and consideration of other possibilities, treatment aiming at normalization of thyroid hormone levels was started and was followed by immediate resolution of symptoms.

With this report, we are documenting a previously unreported case of response to treatment intervention in a well-established phenomenon. There is a clear clinical demonstration of the physiologic-biochemical effect of thyroid hormones both directly and systemically on the kidney that has only been well studied in animal models.

Thyroid hormone replacement should be considered in all such patients of CDI manifesting with low urine output and persistent hypernatremia despite correction with desmopressin. Understanding this mechanism will help in timely diagnosis, ensure improved management, and reduce patient distress and burden of prolonged hospital stay. Apart from the importance of integration into patient care, there is a directive toward future research and clinical endeavors.
